# Identification of Polycystin-2 and CFTR common targets

**DOI:** 10.1186/2046-2530-4-S1-P4

**Published:** 2015-07-13

**Authors:** M Roxo-Rosa, SS Lopes

**Affiliations:** 1 CEDOC - Chronic Diseases Research Center, NOVA Medical School / Faculdade de Ciências Médicas, Universidade Nova de Lisboa, Lisbon, Portugal

## 

Autosomal-Dominant-Polycystic-Kidney-Disease type-II (ADPKD-II) is caused by mutations in the Polycystin-2 (PC2) encoding gene. The dysfunction of this Ca^2+^-conducting channel leads to the formation of fluid-filled renal cysts (1). Through poorly known mechanisms, cystogenesis entails an overstimulation of Cystic-Fibrosis-Transmembrane-conductance-Regulator (CFTR), a key Cl^-^-channel in epithelia's ion/fluid transport (2). To study PC2-CFTR crosstalk we used the zebrafish embryonic node (Kupffer's Vesicle, KV) as a model system. Both proteins are required for KV proper function (3,4).

## Objective

To determine common gene targets of PC2 and CFTR specific knockdown.

## Methods

*foxj1a:gfp *transgenic zebrafish embryos (5) were injected with antisense morpholinos against *pc2 *(augMO-*pc2*) or *cftr *(augMO-*cftr*). This strain offers a KV specific GFP-reporter at 10-11 somites stage. KV cells were isolated by Fluorescent-Activated-Cell-Sorting (FACSAria High-Speed Cell Sorter, BD). Cells from non-injected and mismatch-MO injected embryos were used as controls. Total RNA was extracted (RNAeasy kit, Qiagen) and tested for its quality (Agilent 2100 Bioanalyzer, Affymetrics). Transcriptomes were assessed with the Zebrafish Gene 1.1 ST Array Strip (Affymetrics).

## Results

~2 ng of each morpholino were required to efficiently reduce the PC2 and CFTR expression. In agreement to the literature (3,4), the augMO-*pc2 *induced curly-up tails and the augMO-*cftr *prevented the proper KV lumen expansion. In both cases laterality defects were observed. We have novel information on differentially transcribed genes that we are validating by qPCR.

## Conclusions

Among the PC2- and CFTR-knockdown overlapping targets, we found genes encoding proteins involved in the Calmodulin-mediated Ca^2+^-signalling. These could be involved in the PC2-CFTR crosstalk.

## Acknowledgements

Supported by FCT-ANR/BEX-BID/0153/2012 grant.
